# The involvement of ADAR1 in chronic unpredictable stress-induced cognitive impairment by targeting DARPP-32 with miR-874-3p in BALB/c mice

**DOI:** 10.3389/fcell.2023.919297

**Published:** 2023-04-12

**Authors:** Yanfang Wang, Yingxin Liu, Ziwei Zhao, Xinyu Wu, Jiabin Lin, Yufei Li, Wei Yan, Yi Wu, Yanfei Shi, Xindi Wu, Ying Xue, Jiaqian He, Shuqi Liu, Xiaonan Zhang, Hong Xu, Yiyuan Tang, Shengming Yin

**Affiliations:** ^1^ College of Basic Medical Sciences, Dalian Medical University, Dalian, China; ^2^ National and Local Joint Engineering Research Center for Drug Research and Development of Neurodegenerative Diseases, Dalian, China; ^3^ College of Health Solutions, Phoenix, AZ, United States

**Keywords:** ADAR1, DARPP-32, chronic unpredictable stress, cognitive impairment, BALB/c mice

## Abstract

**Introduction:** Chronic stress exposure is the main environmental factor leading to cognitive impairment, but the detailed molecular mechanism is still unclear. Adenosine Deaminase acting on double-stranded RNA1(ADAR1) is involved in the occurrence of chronic stress-induced cognitive impairment. In addition, dopamine and Adenosine 3′5′-monophosphate-regulated phospho-protein (DARPP-32) gene variation affects cognitive function. Therefore, we hypothesized that ADAR1 plays a key role in chronic stress-induced cognitive impairment by acting on DARPP-32.

**Methods:** In this study, postnatal 21-day-old male BALB/c mice were exposed to chronic unpredictable stressors. After that, the mice were treated with ADAR1 inducer/inhibitor. The cognitive ability and cerebral DARPP-32 protein expression of BALB/c mice were evaluated. In order to explore the link between ADAR1 and DARPP-32, the effects of ADAR1 high/low expression on DARPP-32 protein expression in vitro were detected.

**Results:** ADAR1 inducer alleviates cognitive impairment and recovers decreased DARPP-32 protein expression of the hippocampus and prefrontal cortex in BALB/c mice with chronic unpredictable stress exposure. In vivo and in vitro studies confirm the results predicted by bio-informatics; that is, ADAR1 affects DARPP-32 expression via miR-874-3p.

**Discussion:** The results in this study demonstrate that ADAR1 affects the expression of DARPP-32 via miR-874-3p, which is involved in the molecular mechanism of pathogenesis in chronic unpredictable stress-induced cognitive impairment. The new findings of this study provide a new therapeutic strategy for the prevention and treatment of stress cognitive impairment from epigenetics.

## Introduction

Chronic stress stressors are the main factors inducing cognitive impairment (McEwen and Sapolsky, 1995; Lupien et al., 2009; Marin et al., 2011; An et al., 2017). In the life span, chronic stress stressors impact the cognitive function of the related brain regions, including the hippocampus and frontal cortex (Lupien et al., 2009). Its pathogenesis is mainly that stressors increase the release of sympathetic adrenal medullary hormone in the acute stage (McEwen and Sapolsky, 1995), promote the release of glucocorticoid through the hypothalamus pituitary adrenal axis in the chronic stage (Lupien et al., 2009; Marin et al., 2011), and also cause increased excitatory amino acid release (McEwen and Sapolsky, 1995). Chronic stressors lead to decreased brain-derived neurotrophic factor (BDNF) expression, resulting in a decreased number of neuronal synapses in the medial prefrontal cortex and hippocampus and finally affecting the function of cognitive-related brain areas, showing cognitive impairment (McEwen and Sapolsky, 1995). In addition, stress exposure is the main cause of a variety of brain diseases, including depression and Alzheimer’s disease, and these brain diseases show cognitive impairment in the late stage (Huang et al., 2009; Bennett and Thomas, 2014; Price and Duman, 2020).

So far, the detailed molecular mechanism of stress-induced cognitive impairment is still unclear. Adenosine deaminase acting on double-stranded RNA1 (ADAR1) is the target molecule sensitive to stressors, which provides a new idea to reveal the molecular mechanism of stress-induced cognitive impairment at the epigenetic level. ADAR1 is abnormally expressed in the brain of mice with exposure to social isolation stress and chronic unpredictable stress (Chen et al., 2016; Yu et al., 2018; Zhang et al., 2021a; Zhang et al., 2021b). In addition, ADAR1 expression is significantly increased in the dorsolateral prefrontal cortex of depressive suicidal patients (Simmons et al., 2010). Re-socialization alleviates spatial and non-spatial cognitive impairments and reverses decreased hippocampal ADAR1 expression in isolated KM mice (Chen et al., 2016). In addition, dopamine and adenosine 3′5′-monophosphate-regulated phospho-protein (DARPP-32) is involved in maintaining cognitive function (Houlihan et al., 2009). Homozygous carriers with allele variants of the DARPP-32 gene showing lower DARPP-32 expression have not only higher recall accuracy of episodic memory but also larger volume of the prefrontal cortex (Persson et al., 2017). Image genetics studies have shown that protein phosphatase 1 regulatory inhibitor subunit 1B (PPP1R1B, Gene ID: 84152) genotype variation encoding DARPP-32 affects the function and gray matter integrity of the dorsolateral region of the prefrontal cortex (Meyer-Lindenberg and Zink, 2007; Curcic-Blake et al., 2012). One of the single nucleotide polymorphisms (SNPs) in the PPP1R1B gene, the rs879606A allele, is associated with episodic memory (Curcic-Blake et al., 2012). Trait anger (Reuter et al., 2009) and reward learning (Frank et al., 2007; Frank et al., 2009) are related to the SNP genotype encoding DARPP-32. In addition, DARPP-32 is also involved in the pathogenesis of mental disorders. In the autopsy study of suicidal schizophrenic patients, it is found that the DARPP-32 protein expression level decreases in the prefrontal cortex (Baracskay et al., 2006; Kunii et al., 2011). DARPP-32 is involved in the pathogenesis of mental diseases by affecting the release of neurotransmitters (Beckler et al., 2003). The studies using mouse models of schizophrenia show that various psychotropic drugs work by selective mutation of the DARPP-32 phosphorylation site and DARPP-32 phosphorylation change (Ishikawa et al., 2007). However, so far, it is still unclear whether ADAR1 is involved in the molecular mechanism of chronic stress-induced cognitive impairment *via* DARPP-32.

In this study, the potential link between ADAR1 and DARPP-32 in chronic unpredictable stress-induced cognitive impairment was investigated. The results confirm that ADAR1 inducer alleviates the cognitive impairment and recovers the decreased DARPP-32 expression of the hippocampus and prefrontal cortex in BALB/c mice with chronic unpredictable stress exposure. Moreover, our results confirm that ADAR1 affects DARPP-32 protein expression *in vitro*. Furthermore, miR-874-3p is the potentially related molecule between ADAR1 and DARPP-32 in bioinformatics analysis results. *In vivo* and *in vitro* studies have confirmed that ADAR1 regulates DARPP-32 protein expression *via* miR-874-3p. The novel findings of this study provide a new theoretical basis for further revealing the detailed molecular mechanism of stress-induced cognitive impairment and a new potential molecular target for the prevention and treatment of stress-induced disorders.

## Materials and methods

### Experimental design *in vivo*

**FIGURE 1:**
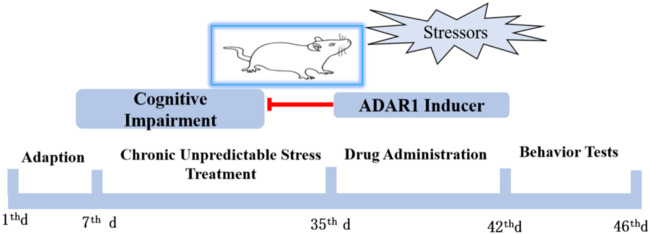
Schedule of the protocol in the experiments *in vivo*. Male BALB/mice were adapted to the standard environment for 1 W. Chronic unpredictable stressors were given to the mice of the model group and drug intervention model group from the 7th day to 35th day. The drug administration was intraperitoneally injected for 1 W. ADARl inducer or ADARl inhibitor was given to the mice of the model group and drug-treated only group. The mice in the control group and model group were intraperitoneally injected with vehicle consecutively. Behavior tests, including new object recognition and new object localization tests, were used to detect the cognitive function of the mice.

Male SPF BALB/c mice weighing 15–20 g and 21 days old were selected. After stable feeding for 1 W in the standard environment of laboratory animal care, the chronic unpredictable stress (CUS) exposure BALB/c mice model was prepared. Behavior tests, including new object recognition and new object localization tests, were used to detect the cognitive function of the mice. According to the principle of random grouping, the experimental mice were divided into six groups, with 13–15 mice in each group: the control group (C), CUS exposure model group (CUS), ADAR1 inducer intervention control group (C + ADAR1 inducer), ADAR1 inducer intervention model group (CUS + ADAR1 inducer), ADAR1 inhibitor intervention control group (C + ADAR1 inhibitor), and ADAR1 inducer intervention model group (CUS + ADAR1 inducer). The drug intervention control group and the drug intervention model group mice were continuously treated with the corresponding ADAR1 targeted drugs for 7 days, and the corresponding behavioral tests were carried out after that. C and CUS group mice were treated with the same dose of solvent to dissolve the drugs. The schedule of the aforementioned experiments is shown in Figure 1. ADAR1, miR-874-3p, and DARPP-32 expressions in the hippocampus and prefrontal cortex of the mice were detected by qPCR and western blot.

### Experimental animals and grouping

Ninety SPF healthy postnatal 21-day-old BALB/c mice were provided by the Animal Experiment Center of Dalian Medical University and Liaoning Changsheng Biotechnology Company. After exclusion, 85 mice were used in this study. Mice with exposure to chronic stressors showing evident decrease in body weight were excluded. The experiment has been approved by the Animal Ethics Committee of Dalian Medical University (L20140021). After 7 days of adaptive feeding, the mice were randomly divided into six groups with 13–15 mice in each group: normal control group (C), chronic unpredictable stress model group (CUS), ADAR1 inducer intervention control group (C + ADAR1 inducer), ADAR1 inhibitor (pentostatin, PEN, or erythro-9-(2-hydroxy-3-nonyl) adenine, EHNA) intervention control group (C + ADAR1 inhibitor), ADAR1 inducer intervention model group (CUS + ADAR1 inducer), and ADAR1 inhibitor intervention model group (CUS + ADAR1 inhibitor).

### Preparation of chronic unpredictable stress mice model

The mice were adapted to the normal feeding environment for 1 W and were given a sufficient diet in the normal feeding environment. The room temperature was maintained at 22°C ± 1°C, and the humidity was 60% ± 5%. A 12 h/12 h light–dark cycle was followed. The noise was ≤20 dB. After that, the BALB/c mice were exposed to various mild stresses, including lighting all night for 10 h, fasting for 24 h, water prohibition for 24 h, single cage feeding for 24 h, tail clamping for 30 min (1 cm from the tail root), forced swimming for 10 min, inclined padding for 5 h, and restraint for 1 h. Two different stressors were given to the experimental mice per day. A stressor was not repeated until 2 days. The aforementioned stressor exposures were administered for 4 W. The mice in the control group lived in the normal feeding environment. Only the mice in the control group were handled every day. After the exposures, the mice were housed in the experimental environment for 1–2 h to avoid the impact of the odor caused by stressors.

### New object recognition and new object location tests

New object recognition and new object localization tests are the classical experiments to evaluate the spatial and non-spatial cognitive functions of experimental animals (An et al., 2017). BALB/c mice distinguish a new object from an old object according to the characteristics and spatial position of the new object and the old object. Before the formal experiment, the experimental mice were put into a transparent experimental box without any object. Each mouse was trained 10 min a day for three consecutive days. In the familiarity period, the same object was placed in the box, the specific position of the object was recorded by video, and the position of the object was marked automatically. After 4 h, in the test period, the objects were put with different appearances or positions in the box, and the operation was the same as that in the familiarity period. The experimental system recorded the exploration time for two different objects in the familiarity period and the test period, respectively. The exploration time was the closed contact of the mouse’s head and nose with the object. The exploration time of each mouse was controlled for 5 min. At the beginning of the experiment, the experimental mice were placed between two objects. The calculation formula of the discrimination index in the test period is as follows: (time to explore new objects − time to explore old objects)/(time to explore new objects + time to explore old objects).

### Drug administration

The drug administration was intraperitoneally injected. The dosage was based on the pilot study and the literature (Zhang et al., 2021a). ADAR1 inducer (interferon-γ, IFN-γ, 2.0 × 10^5^ U/kg/d, i.p. q.d. BioLegend Biotechnology, China) was administered to the mice of the ADAR1 inducer intervention control group and ADAR1 inducer intervention model group. ADAR1 inhibitors PEN (1 mg/kg/d, i.p. q.d. MCE, United States) or EHNA (10 mg/kg/d, i.p. q.d. Sigma, United States) were administered to the mice of the ADAR1 inhibitor intervention control group and ADAR1 inhibitor intervention model group, respectively. The drug concentration of PEN was 1 mg/mL, and the drug concentration of EHNA was 0.5 mg/mL. The mice in the control group and model group were intraperitoneally injected with normal saline at a dose of 20 mL/kg/d for 7 days consecutively. In the murine adrenal medullary pheochromocytoma (PC12) cell line, ADAR1 inducer (IFN-γ, 50 ng/mL) or ADAR1 inhibitor (EHNA, 0.2 mmol/L) was administered.

### Preparation of ADAR1 high/low expression cell line

PC12 cells were cultured in a 6-cm dish to 80%–90% fusion, the culture medium was removed, and the cells were washed twice with 1 mL PBS. Next, 1 mL of DMEM culture solution containing 10% fetal bovine serum (FBS) was added to 200 μL pancreatin. The cells were fully mixed in the aforementioned treatment and were placed for 1–2 min. Then, 200 μL culture medium was added to stop digestion, and blowing was performed to cause cells to form a single-cell suspension. Centrifugation at 1,000 rpm for 5 min was performed, then the supernatant sample was discarded, and 1 mL culture medium was added to re-suspend the cells. Four small dishes were prepared. Then, 1.5 mL DMEM culture solution containing serum was added to each dish, and 250 μL fully mixed cell suspension was added to each dish. The four plates of cells in the incubator were placed with 5% CO_2_ at 37°C. After 12 h, when the cell density reached about 70%, transfection was carried out. A 600-μL sterile centrifuge tube was prepared, with 100 μL serum-free DMEM in each tube. The transfection reagent in the four tubes was added according to the instructions of the GP-transfect-Mate transfection reagent, was mixed gently using a pipette, and was kept at room temperature for 5 min; at the same time, the corresponding amount of RNA oligo/DNA was added into the other four tubes, was gently mixed using a pipette, and was kept at room temperature for 5 min. The mixture of the GP-transform-Mate medium was added to the mixture of the RNA oligo/DNA medium, was mixed gently using a pipette, and was kept at room temperature for 20 min. The solutions of the four small dishes were changed, and the content of each dish was replaced with 1 mL of preheated DMEM containing 3% FBS. The transfection mixture was added to the corresponding dish, and the final system was 500 μL. The small dishes were shaken gently to evenly distribute the compound. The cells were incubated at 37°C and then changed into DMEM containing 10% FBS after 5 h. PC12 cell lines were divided into the following groups: control group, negative empty vector transfection group, ADAR1 (GeneID: Adar, 56417) high-expression transfection group, and ADAR1 low-expression transfection group. The transfection was carried out according to the manufacturer’s instructions (Suzhou Jima Gene Co., Ltd.).

### Bioinformatics analysis

In order to analyze the potential molecular mechanism of ADAR1 affecting DARPP-32 protein expression, bioinformatics analysis was used to analyze the potentially related molecules between ADAR1 and DARPP-32 proteins. Mmu-microRNA molecules potentially related to ADAR1 and DARPP-32 are predicted through “TargetScanHuman.” According to the characteristics of A-I editing, the stem–loop structure of mmu-microRNA molecules potentially related to ADAR1 and DARPP-32 were analyzed with “RNAfold WebServer.” Then, the binding sites of the target mmu-microRNA molecules with ADAR1 mRNA and DARPP-32 mRNA were predicted through “TargetScanHuman.”

### qRT-PCR assay

In total, 50 mg mouse brain tissue was added to a 5-mL sterile centrifuge tube, 1 mL RNA extract TRIzol was added into the tube, and then the tube was placed on ice for precooling. Then, the sample was fully homogenized with a homogenizer and was left on ice for 5 min. The cells were digested in each dish with pancreatin and sucked into a 1.5-mL sterile centrifuge tube. Then, they were centrifuged at 1,000 rpm for 5 min and washed twice with PBS. The supernatant was discarded, and cell precipitation was collected. Then, 1 mL of RNA extract TRIzol was added to each tube. A pipette gun was used to repeatedly and forcefully aspirate to make the cell lyse, and then the sample was kept at room temperature for 5 min. The tissue and cell samples were treated as follows: 200 µL chloroform was added to each tube, was mixed upside down, was placed on ice for 5 min, and was then centrifuged at 12,000 *g* 4°C for 15 min. The upper colorless water phase was taken into a new 1.5-mL sterile centrifuge tube, and 400 µL isopropanol was added. Then, the content was mixed upside down, was placed on the ice for 10 min, and was centrifuged at 12,000 *g* 4°C for 15 min. The supernatant was discarded, and colloidal sediment was obtained. Next, 75% ethanol with anhydrous ethanol and DEPC water was prepared, 500 µL of 75% ethanol was added into each tube, and the tube was turned upside down to make the sediment float. After centrifugation at 4°C for 10 min, the supernatant was discarded. This operation was repeated, and the sediment was washed again. The residual alcohol on the tube wall was absorbed by filter paper, and 10 µL of DEPC water was added into each tube to dissolve RNA after the precipitation was fully dried. A NanoDrop ultraviolet spectrophotometer was used to measure RNA concentration. The ratio of A260/A280 was between 1.8–2.0. The samples were stored at −80°C for use. The primer sequences of qRT-PCR are shown in Table 1. After mixing the RNA sample with the reverse transcription reagent according to the instructions of the Akeri AG11711 reverse transcription kit (Accurate, China), the two-step method was used to remove the genomic DNA at 42°C for 2 min and then recovered to 4°C. Then, the reverse transcription reaction was carried out at 37°C for 15 min, 85°C for 5 s, and then the sample was stored at 4°C. After mixing the cDNA with the system according to the instructions of the Akeri AG11701qPCR kit (Accurate, China), the two-step method was used. The first step was 95°C for 30 s; the second step was 95°C for 5 s, 60°C for 30 s, 40 cycles; and the third step was to add the dissolution curve. After the reaction, the Ct value was obtained, and the 2^-∆∆t^ value was calculated and analyzed.

**TABLE 1:** Primer sequence of qRT-PCR.

Target molecule	Forward primer (5′to 3′)	Reverse primer (5′to 3′)
^1^rno-miR-874-3p	TAATGCTGCTGCCCTGGC	TAT​GGT​TGT​TCA​CGA​CTC​CTT​CAC
^2^rno-ADAR1	GGT​GCT​TGG​CTG​ATG​GCT​ATG​AC	CAA​ATC​TCT​GCG​GGC​TCG​GAA​G
^2^rno-Darpp-32	CCA​TCA​GCA​ACC​TGA​GTG​AGA​ACC	CGT​CCC​TCT​TCA​TCC​TCG​TCC​TC
^2^rno-GAPDH	GAC​ATG​CCG​CCT​GGA​GAA​AC	AGC​CCA​GGA​TGC​CCT​TTA​GT
^1^U6	CGC​TTC​GGC​AGC​ACA​TAT​AC	TTC​ACG​AAT​TTG​CGT​GTC​ATC
^1^MmiR-874-3p	TAATGCTGCTGCCCTGGC	TAT​GGT​TGT​TCA​CGA​CTC​CTT​CAC
^2^mmu-ADAR1	AGC​CAC​AGG​TGC​TTC​AAT​GC	GTC​CCC​TTT​CAC​ACA​GCG​ATT
^2^mmu-Darpp-32	AGA​TTC​AGT​TCT​CTG​TGC​CCG	GGT​TCT​CTG​ATG​TGG​AGA​GGC
^2^mmu-GAPDH	GCC​ACC​CAG​AAG​ACT​GTG​GAT	GGAAGGCCATGCCAGTGA

1, Gene Pharma, China; 2, Wanze, China.

### Western blot

The mice were anesthetized with 4% isoflurane, the brain tissue was isolated, and the prefrontal cortex and hippocampus tissue were selected according to the localization of the brain atlas, respectively. Lysis buffer was added into the tube containing the brain tissue (100 mg/0.5 mL), and the sample was fully homogenized at low temperature 30–50 times until the tissue blocks were unrecognizable by the naked eye. Then, the sample was fully centrifuged at 4°C for 5 min at 12,000 rpm, the supernatant was taken off, and the cerebral samples were collected for standby. The cell line samples were also collected for standby. The protein was extracted later. Then, 10 μL phosphatase inhibitor, 1 μL protease inhibitor, and 10 μL 100 mm phenylmethanesulfonyl fluoride (PMSF) lysis buffer were added to 1 mL cold lysis buffer, respectively. Then, they were fully mixed and put on ice for standby. The protein concentration was measured using the BCA method. Electrophoresis and membrane transfer were performed. After blocking, the polyvinylidene fluoride (PVDF) membrane was cleaned with Tris-buffered saline with Tween (TBST) buffer. The membrane was washed three times for 10 min each time. Then, primary antibody ADAR1 (1:1,000, Proteintech, China), DARPP-32 (1:1,000, Huabio, China), and *ß*-actin (1:5,000, Bioss, China) were added. The PVDF membrane was cultured with the primary antibody at 4°C overnight. After recovering the primary antibody, the PVDF membrane was washed with TBST buffer three times for 10 min each time. The secondary antibody, goat anti-rabbit IgG/HRP (1:10,000, cell signaling technology, United States), was cultured with the membrane. The sample was slowly shaken in the shaker for 2 h at room temperature. The hypersensitive luminescent liquid was prepared with A and B (1:1) solution in the dark room. The PVDF membrane was washed with TBST buffer three times for 10 min each time. The hypersensitive luminescent liquid was uniformly distributed onto the PVDF film. The gray values of the target protein were analyzed with the gel imaging system.

### Statistical analysis

SPSS 23.0 (Aramonk, NY, United States) and GraphPad 7.0 (San Diego, CA, United States) were used for statistical analysis. The data were compared between the two groups and multiple groups by *t*-test and two-way ANOVA, respectively. Two-way ANOVA was used to determine whether there is an interaction between chronic unpredictable stress and ADAR1 target intervention (two independent variables) on DARPP-32 protein expression (dependent variable) among the mice. The *t*-test was used to analyze the variance for the groups with and without CUS exposure, the groups with and without ADAR1 target intervention in the mice, and the groups with and without transfection in PC 12 cell lines. All data were expressed as the mean average value ± standard difference (mean ± standard deviation), and *p* < 0.05 was expressed as a significant difference.

## Results

ADAR1 inducer alleviates spatial and non-spatial cognitive impairment in mice exposed to chronic unpredictable stressors.

**FIGURE 2:**
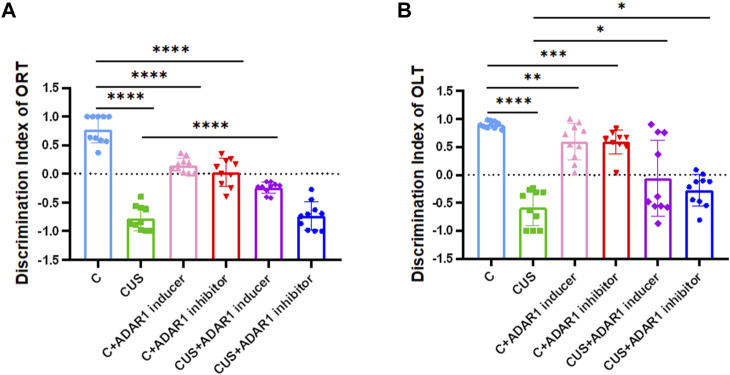
ADARl inducer alleviates spatial and non-spatial cognitive impairment in mice exposed to chronic unpredictable stressors. **(A)** Discrimination index of the mice in the new object recognition test (ORT). **(B)** Discrimination index of the mice in the new object location test (OLT); “C” represents normal control group, “CUS” represents chronic unpredictable stressors-treated model group, “C + ADARl inhibitor” represents ADARl inhibitor (PEN) intervention control group, “C + ADARl inducer” indicates ADARl inducer (INF-γ) intervention control group, “CUS + ADAR1 inducer” represents ADARI inducer intervention model group, and “CUS + ADARl inhibitor” indicates ADARl inhibitor intervention model group. *p < 0.05, **p < 0.01, ***p < 0.001, ****p < 0.0001, (n = 10/group). The data are expressed as mean ± standard deviation.

In the new object recognition test, ADAR1 inducer reversed non-spatial cognitive impairment in BALB/c mice exposed to chronic unpredictable stressors(Figure 2). As compared with that in the control group, the discrimination index of the mice in the model group decreased significantly (C: 0.78 ± 0.08, CUS: −0.78 ± 0.07, *p* < 0.0001); compared with that of the model group, the discrimination index of the mice in the ADAR1 inducer-treated model group increased significantly (CUS: −0.78 ± 0.07, CUS + ADAR1 inducer: −0.24 ± 0.03, *p* < 0.0001). There is no significant difference between the discrimination index of the mice in the model group and ADAR1 inhibitor-treated model group. These results suggest that chronic unpredictable stress exposures lead to non-spatial cognitive impairment in BALB/c mice, and ADAR1 inducer alleviates non-spatial cognitive impairment in BALB/c mice exposed to chronic unpredictable stressors. In addition, as compared with the control group (0.78 ± 0.08), the discrimination index of the mice in both the ADAR1 inducer-treated only group (0.15 ± 0.04) or ADAR1 inhibitor-treated only group (0.03 ± 0.08) decreased evidently (both *p* < 0.0001), which suggest that the breaking of ADAR1 homeostasis leads to non-spatial cognitive impairment.

In the new object localization test, ADAR1 inducer reversed spatial cognitive impairment in the BALB/c mice exposed to chronic unpredictable stressors. As compared with that in the control group, the discrimination index of the mice in the model group decreased significantly (C: 0.90 ± 0.02, CUS: −0.59 ± 0.10, *p* < 0.0001); compared with that of the model group, the discrimination index of the mice in the ADAR1 inducer-treated model group increased significantly (CUS: −0.59 ± 0.10, CUS + ADAR1 inducer: −0.06 ± 0.22, *p* < 0.05); compared with that of the model group, the discrimination index of the mice in the ADAR1 inhibitor-treated model group increased significantly (CUS: −0.59 ± 0.10, CUS + ADAR1 inhibitor: −0.27 ± 0.09, *p* < 0.05). These results suggest that chronic unpredictable stress exposures lead to spatial cognitive impairment in BALB/c mice and that ADAR1 inducer alleviates the spatial cognitive impairment. Interestingly, ADAR1 inhibitor does not aggravate the spatial cognitive impairment in BALB/c mice exposed to chronic unpredictable stressors. This may also be related to the difference between the brain regions involved in spatial cognitive and non-spatial cognitive functions (Long and Zhang, 2021) and compensatory mechanisms. In addition, as compared with the control group (0.90 ± 0.02), the discrimination index of the mice in both the ADAR1 inducer-treated only group (0.59 ± 0.10, *p* < 0.01) or ADAR1 inhibitor-treated only group (0.59 ± 0.07, *p* < 0.0001) decreased evidently, which suggest that the breaking of ADAR1 homeostasis leads to the spatial cognitive impairment.

### ADAR1 inducer reverses low cerebral DARPP-32 protein expression of the cognitive impairment in mice

**FIGURE 3:**
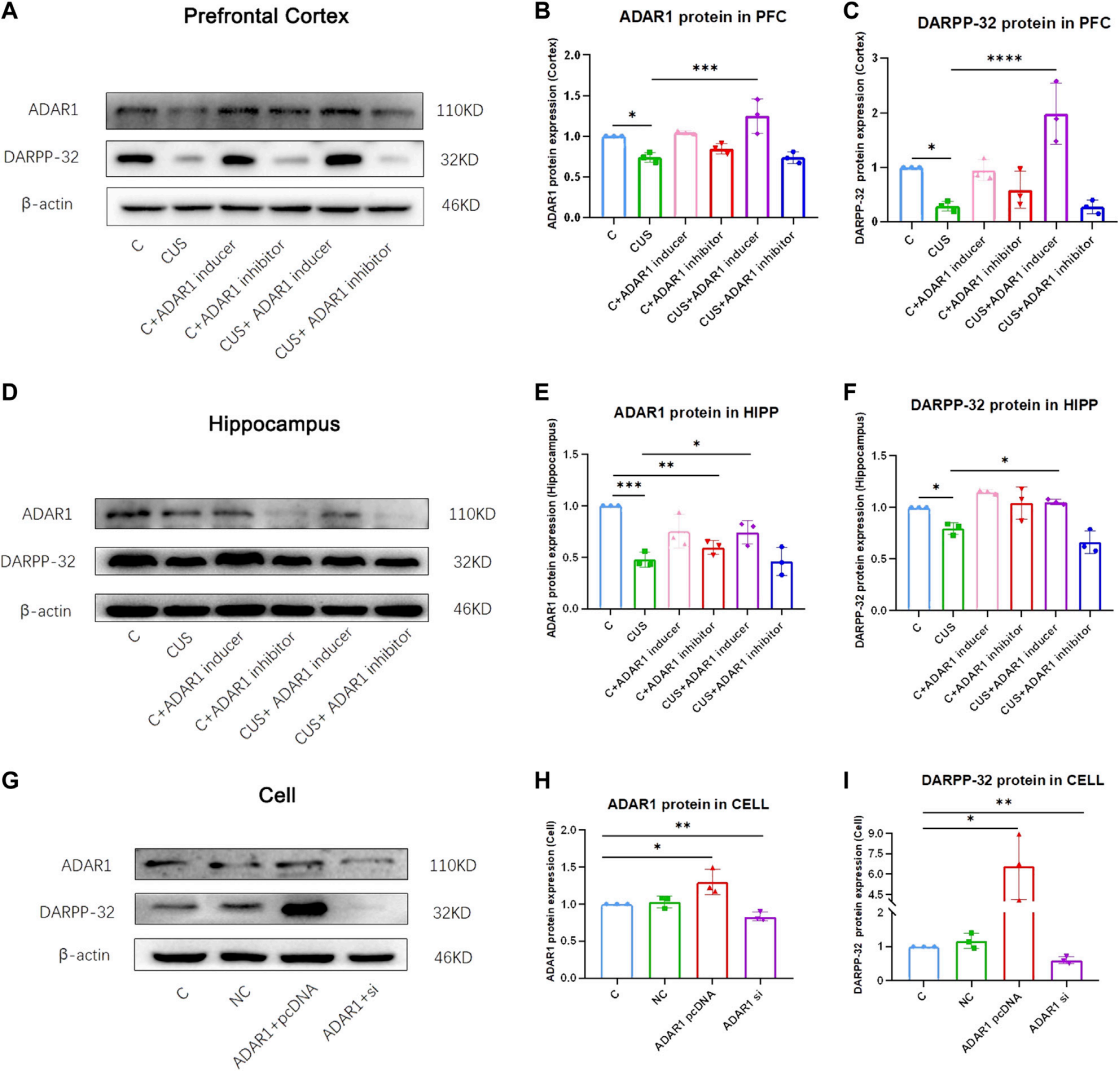
ADAR1 impacts DARPP32 protein expression *in vivo* and *in vitro*. **(A)** ADAR1 and DARPP-32 protein expression in prefrontal cortex of the mice. **(B)** Gray value analysis of ADARl protein expression in the prefrontal cortex of the mice. **(C)** Gray value analysis of DARPP-32 protein expression in the prefrontal cortex of the mice. **(D)** ADAR1 and DARPP-32 protein expressions in the hippocampus of the mice. **(E)** Gray value analysis of ADARl protein expression in the hippocampus of the mice. **(F)** Gray value analysis of DARPP-32 protein expression in hippocampus of the mice. “C” represents normal control group, “CUS” represents chronic unpredictable stressors treated model group, “C + ADAR1 inhibitor” represents the ADARl inhibitor (PEN/EHNA) intervention control group; EHNA was used in the results of **(A-I)**, “C + ADARl inducer” indicates the ADARl inducer (IFN-γ) intervention control group, “CUS + ADAR1 inducer” represents the ADARl inducer intervention model group, and “CUS + ADAR1 inhibitor” indicates the ADARl inhibitor intervention model group. **(G)** ADARl and DARPP-32 protein expressions in ADARl over/low-expression PC12 cell lines. **(H)** Gray value analysis of ADARl protein expression in ADARl over/low-expression PC12 cell lines. **(I)** Gray value analysis of DARPP-32 protein expression in ADARl over/low-expression PC12 cell lines. “C” represents the normal control group, “NC” represents the negative empty vector group, “ADARl pcDNA” represents the ADARl over-expression group, and “ADARl si” represents the ADARl low-expression group. *p < 0.05, **p < 0.01, ***p < 0.001, ****p < 0.0001, (*n* = 3/group). Data are expressed as mean ± standard deviation.

ADAR1 inducer/inhibitor intervention impacts DARPP-32 protein expression in the prefrontal cortex and hippocampus of BALB/c mice exposed to chronic unpredictable stressors (Figures 3A–F). In the prefrontal cortex, the expression of DARPP-32 protein in the mice exposed to chronic unpredictable stressors decreased significantly as compared with that of the control mice (C: 1.00 ± 0.00, CUS: 0.29 ± 0.09, *p* < 0.05); meanwhile, the expression of ADAR1 protein in the mice exposed to chronic unpredictable stressors decreased significantly as compared with that of the control mice (C: 1.00 ± 0.00, CUS: 0.74 ± 0.06, *p* < 0.05). In addition, as compared with that of the chronic unpredictable stress model group, DARPP-32 protein expression of the mice in the ADAR1 inducer intervention chronic unpredictable stress model group increased significantly as compared with that of the model group (CUS: 0.29 ± 0.09, CUS + ADAR1 inducer: 1.98 ± 0.56, *p* < 0.0001); meanwhile, ADAR1 protein expression of the mice in the ADAR1 inducer intervention chronic unpredictable stress model group increased significantly as compared with that of the model group (CUS: 1.98 ± 0.56, *p* < 0.0001 CUS + ADAR1 inducer: 1.98 ± 0.56, *p* < 0.0001). These results suggest that ADAR1 inducer reversed the decreased DARPP-32 and ADAR1 protein expressions in the prefrontal cortex of the mice in the chronic unpredictable stress model group. In the hippocampus, the expression of DARPP-32 protein in the mice exposed to chronic unpredictable stressors decreased significantly as compared with that of the control mice (C: 1.00 ± 0.00, CUS: 0.80 ± 0.06, *p* < 0.05); meanwhile, the expression of ADAR1 protein in the mice exposed to chronic unpredictable stressors decreased significantly as compared with that of the control mice (C: 1.00 ± 0.00, CUS: 0.48 ± 0.07, *p* < 0.05). In addition, as compared with that of the chronic unpredictable stress model group, DARPP-32 protein expression of the mice in the ADAR1 inducer intervention chronic unpredictable stress model group increased significantly as compared with that of the model group (CUS: 0.80 ± 0.06, CUS + ADAR1 inducer: 1.05 ± 0.03, *p* < 0.05); meanwhile, ADAR1 protein expression of the mice in ADAR1 inducer intervention chronic unpredictable stress model group increased significantly as compared with that of the model group (CUS: 0.48 ± 0.07, CUS + ADAR1 inducer 0.74 ± 0.12, *p* < 0.05). These results suggest that ADAR1 inducer reversed the decreased DARPP-32 and ADAR1 protein expressions in the hippocampus of the mice in the chronic unpredictable stress model group.

### Over-expression and low-expression of ADAR1 affect DARPP-32 protein expression

In PC12 cell lines, over-expression or low-expression of ADAR1 affects DARPP-32 protein expression (Figures 3G–I). In western blot, as compared with that of the control group, the DARPP-32 (C: 1.00 ± 0.00, ADAR1 pcDNA: 6.58 ± 2.43 *p* < 0.05) and ADAR1 (C: 1.00 ± 0.00, ADAR1 pcDNA:1.29 ± 0.17, *p* < 0.05) protein expressions in the ADAR1 over-expression group were significantly increased; meanwhile, DARPP-32 (C: 1.00 ± 0.00, ADAR1 si: 0.60 ± 0.10, *p* < 0.01) and ADAR1 (C: 1.00 ± 0.00, ADAR1 si: 0.83 ± 0.06, *p* < 0.01) protein expressions in the ADAR1 low-expression group were decreased significantly. These results suggest that ADAR1 impacts DARPP-32 protein expression *in vitro*.

### The potential microRNA related to DARPP-32 evaluated by bioinformatics

The potential microRNAs related to DARPP-32 were analyzed by “TargetScanHuman.” The results show that mouse-derived microRNAs related to DARPP-32 include mmu-miR-6240, mmu-miR-330-3p.2, mmu-miR-1941-5p, mmu-miR-6913-5p, mmu-miR-3474, mmu-miR-3572-5p, and mmu-874-3p. Mouse-derived microRNAs related to ADAR1 include mmu-miR-1a-3p, mmu-miR-206-3p, mmu-miR-6349, mmu-miR-1957b, mmu-miR-206-3p, mmu-miR-6382, and mmu-874-3p. Mmu-874-3p is associated with both DARPP-32 and ADAR1.

The stem–loop structures of mmu-pre-miR-874 were analyzed by RNAfold WebServer (http://rna.tbi.univie.ac.at//cgi-bin/RNAWebSuite/RNAfold.cgi). The pre-mRNA of mmu-pre-miR-874 has potential sites edited by A-I. According to the analysis using “TargetScanHuman,” mmu-874-3p has seven binding sites between site 316 and site 322 in 3′UTR of DARPP-32 mRNA. Mmu-874-3p has seven binding sites between site 1,092 and site 1,098 in 3′UTR of ADAR1 mRNA. According to the aforestated analysis results, ADAR1 is supposed to regulate DARPP-32 expression *via* miR-874-3p (Figure 5).

### ADAR1 impacts DARPP-32 expression *via* miR-874-3p

**FIGURE 4:**
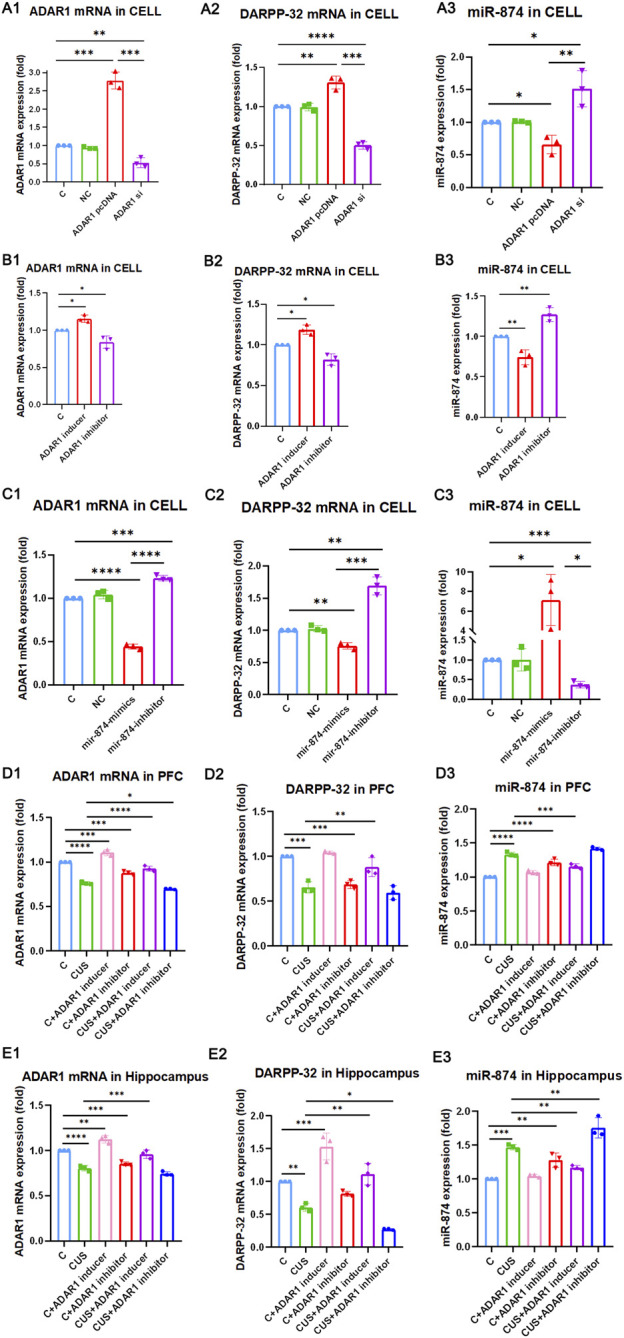
ADAR1 impacts DARPP-32 expression *via* miR-874-3p *in vivo* and *in vitro*. **(A1-A3)** ADAR1 high/low (A1) expression impacts DARPP-32 mRNA (A2) and miR-874-3p (A3) expression, respectively. **(B1-B3)** ADAR1 inducer (IFN-γ) and ADAR1 inhibitor (EHNA) impact ADAR1 mRNA (B1) DARPP-32 mRNA (B2) and miR-874-3p (B3) expression, respectively. **(C1-C3)** miR-874-3p high/low (C3) expression impacts ADAR1 (C1) and DARPP-32 (C2) mRNA expression, respectively. **(D1-D3)** ADAR1 inducer (IFN-γ) evidently recovered decreased ADAR1 mRNA (D1) and DARPP-32 mRNA (D2) and increased miR-874-3p (D3) expression induced by chronic unpredictable stressors, respectively, in the prefrontal cortex; meanwhile, ADAR1 inhibitor (EHNA) evidently aggravated decreased ADAR1 mRNA (D1) and showed the decreased tendency of DARPP-32 mRNA (D2) and increased tendency of miR-874-3p (D3) expression induced by chronic unpredictable stressors, respectively, in the prefrontal cortex. **(E1-E3)** ADAR1 inducer (IFN-γ) evidently recovered decreased ADAR1 mRNA (E1), decreased DARPP-32 mRNA (E2), and increased miR-874 (E3) expression induced by chronic unpredictable stressors, respectively, in the hippocampus; meanwhile, ADAR1 inhibitor (EHNA) evidently aggravated decreased DARPP-32 mRNA (E2) and increased miR-874 (E3) expression and also showed the decreased tendency of ADAR1 mRNA expression (E1) induced by chronic unpredictable stressors, respectively, in the hippocampus.

To further verify whether ADAR1 affects the expression of DARPP-32 through miR-874-3p, first, in PC12 cell lines with high/low expression of ADAR1, the effects of DARPP-32 and miR-874-3p mRNA expression were observed (Figure 4 A1-3). The results showed that when ADAR1 was highly expressed, ADAR1 mRNA expression increased (C: 1.00 ± 0.00, ADAR1 pcDNA: 2.78 ± 0.23, *p* < 0.001), DARPP-32 mRNA expression also increased (C: 1.00 ± 0.00, ADAR1 pcDNA: 1.31 ± 0.08, *p* < 0.01), while miR-874-3p expression decreased (C: 1.00 ± 0.00, ADAR1 pcDNA: 0.66 ± 0.14, *p* < 0.05). In addition, when ADAR1 expression was low (C: 1.00 ± 0.00, ADAR1 si: 0.53 ± 0.13, *p* < 0.01), DARPP-32 mRNA expression also decreased (C: 1.00 ± 0.00, ADAR1 si: 0.50 ± 0.05, *p* < 0.01), while miR-874-3p expression increased (C: 1.00 ± 0.00, ADAR1 si: 1.51 ± 0.28, *p* < 0.05). Then, ADAR1 inducer or inhibitor was intervened in the PC12 cell line (Figure 4 B1-3), and results similar to those of the *in vivo* experiment were obtained. ADAR1 mRNA increased in the ADAR1 inducer group (C: 1.00 ± 0.00, ADAR1 inducer: 1.15 ± 0.05, *p* < 0.05), the expression of DARPP-32 mRNA also increased (C: 1.00 ± 0.00, ADAR1 inducer: 1.19 ± 0.06, *p* < 0.05), but the expression of miR-874-3p decreased (C: 1.00 ± 0.00, ADAR1 inducer: 0.74 ± 0.09, *p* < 0.01); ADAR1 mRNA decreased in the ADAR1 inhibitor group (C: 1.00 ± 0.00, ADAR1 inhibitor: 0.84 ± 0.09, *p* < 0.05), the expression of DARPP-32 mRNA also decreased (C: 1.00 ± 0.00, ADAR1 inhibitor: 0.82 ± 0.07, *p* < 0.05), but the expression of miR-874-3p increased (C: 1.00 ± 0.00, ADAR1 inhibitor: 1.27 ± 0.09, *p* < 0.01);

Then, the results in PC12 cell lines with miR-874-3p high/low expression showed that miR-874 expression increased in the miR-874 mimics group (C: 1.00 ± 0.00, miR-874 mimics: 7.11 ± 2.62, *p* < 0.05) and that miR-874 expression decreased in the miR-874 inhibitor group (C: 1.00 ± 0.00, miR-874 inhibitor: 0.37 ± 0.08, *p* < 0.001). Then, the expressions of DARPP-32 and ADAR1 mRNA were also observed (Figure 4 C1-3). The results showed that miR-874-3p high expression reduced DARPP-32 mRNA expression (C: 1.00 ± 0.00, miR-874 mimics: 0.76 ± 0.05, *p* < 0.01) and ADAR1 mRNA expression (C: 1.00 ± 0.00, miR-874 mimics: 0.44 ± 0.03, *p* < 0.0001). In addition, low miR-874-3p expression increased DARPP-32 mRNA expression (C: 1.00 ± 0.00, miR-874 inhibitor: 1.69 ± 0.14, *p* < 0.01) and ADAR1 mRNA expression (C: 1.00 ± 0.00, miR-874 inhibitor: 1.23 ± 0.03, *p* < 0.001). The *in vivo* results (Figure 4 D1-3 and E1-3) showed that as compared with that in the control group, the expressions of ADAR1 mRNA and DARPP-32 mRNA were significantly decreased in the hippocampus [ADAR1: (C: 1.00 ± 0.00, CUS: 0.80 ± 0.03, *p* < 0.0001); DARPP-32: (C: 1.00 ± 0.00, CUS: 0.61 ± 0.06, *p* < 0.01)] and frontal cortex [ADAR1: (C: 1.00 ± 0.00, CUS: 0.76 ± 0.02, *p* < 0.0001); DARPP-32: (C: 1.00 ± 0.00, CUS: 0.66 ± 0.06, *p* < 0.001)] of the model group mice, while miR-874-3p expression was significantly increased in the hippocampus (C: 1.00 ± 0.00, CUS: 1.47 ± 0.04, *p* < 0.001) and frontal cortex (C: 1.00 ± 0.00, CUS: 1.33 ± 0.03, *p* < 0.0001); meanwhile, ADAR1 inducer reversed the decreased ADAR1 mRNA and DARPP-32 mRNA expressions in the hippocampus [ADAR1: (CUS: 0.80 ± 0.03, CUS + ADAR1 inducer: 0.96 ± 0.05, *p* < 0.001); DARPP-32: (CUS: 0.61 ± 0.06, CUS + ADAR1 inducer: 1.11 ± 0.17, *p* < 0.01)] and frontal cortex [ADAR1: (CUS: 0.76 ± 0.02, CUS + ADAR1 inducer: 0.93 ± 0.03, *p* < 0.0001); DARPP-32: (CUS: 0.66 ± 0.06, CUS + ADAR1 inducer: 0.88 ± 0.10, *p* < 0.01)]. Moreover, ADAR1 inducer reversed the increased miR-874-3p expression in the hippocampus (CUS: 1.47 ± 0.04, CUS + ADAR1 inducer: 1.12 ± 0.03, *p* < 0.01) and frontal cortex (CUS: 1.33 ± 0.03, CUS + ADAR1 inducer: 1.16 ± 0.04, *p* < 0.001) induced by chronic stressors. In addition, ADAR1 inhibitor aggravated the decreased ADAR1 mRNA expression in the frontal cortex (CUS: 0.76 ± 0.02, CUS + ADAR1 inhibitor: 0.70 ± 0.01, *p* < 0.05), aggravated the decreased DARPP-32 mRNA expression in the hippocampus (CUS: 0.61 ± 0.06, CUS + ADAR1 inhibitor: 0.27 ± 0.01, *p* < 0.05), and aggravated the increased miR-874-3p expression in the hippocampus (CUS: 1.47 ± 0.04, CUS + ADAR1 inhibitor: 1.76 ± 0.15, *p* < 0.01) induced by chronic stressors. The aforestated results demonstrate that ADAR1 impacts DARPP-32 expression *via* miR-874-3p.

## Discussion

It has been confirmed that both humans and animals show cognitive impairment after exposure to chronic stressors in different life spans (McEwen and Sapolsky, 1995; Lupien et al., 2009; Marin et al., 2011). In human studies, it is found that exposure to stressors in the perinatal period, childhood, adolescence, and adulthood cause varying degrees of cognitive impairments (Marin et al., 2011). Adolescents who continue to suffer from adversity in the early stage show shrinkage of the anterior cingulate cortex and reduction in gray matter volume of the frontal cortex (Cohen et al., 2006). In human beings, the frontal cortex continues to develop in adolescence and is vulnerable and sensitive to stressors (Lupien et al., 2009). In animal studies, rats exposed to prenatal stressors show learning disabilities in adulthood (Vallee et al., 1999). According to research studies, 3-week-old KM mice and BALB/c mice show cognitive deficits after exposure to social isolation stress; meanwhile, re-socialization alleviates the aforementioned cognitive impairments (Chen et al., 2016). C57BL/6J mice exposed to chronic stressors in adulthood cause dendritic retraction of pyramidal neurons in the prefrontal cortex area II/III and decreased number of dendritic spines (Izquierdo et al., 2006). It should be noted that when humans and animals are in puberty, their brains are more easily impacted by the stressors and high levels of glucocorticoids than in adulthood, which is related to the fact that the negative feedback mechanism of the HPA axis has not been fully established (Izquierdo et al., 2006). At present, the mechanism of stress-induced cognitive impairment is mainly due to the abnormal function of the HPA axis leading to the excessive glucocorticoids release, and the glucocorticoids act on the glucocorticoid receptor in the frontal cortex, which is closely related to cognitive function. During chronic stress exposure, glucocorticoid expression increases, and glucocorticoid receptors (GR or type II) are distributed in the prefrontal cortex mainly (McEwen et al., 1986; Diorio et al., 1993; Sanchez et al., 2000). The release of glucocorticoids is regulated by the feedback of the HPA axis, and the prefrontal cortex and hippocampus play an inhibitory role in the HPA axis (Dunn and Orr, 1984; Herman et al., 2005). It has been confirmed that higher cortisol levels are positively correlated with an increase in cognitive decline (Weiner et al., 1997; Umegaki et al., 2000; Huang et al., 2009). However, so far, the detailed molecular mechanism of stress-induced cognitive impairment has not been fully clarified. Environmental stress exposures affect animal behavior *via* the epigenetic regulation mechanism (Chen et al., 2016; Yu et al., 2018; Zhang et al., 2021a; Zhang et al., 2021b), which provides a new idea to reveal the mechanism of stress-induced cognitive impairment.

It is known that chronic social isolation stress exposure causes cognitive decline in KM mice, accompanied by an increased ADAR1 expression in the hippocampus and cortex, and these abnormal manifestations can be reversed by re-socialization (Chen et al., 2016). In addition, ADAR1 expression in the frontal cortex, hippocampus, and amygdala of KM mice and BALB/c mice exposed to social isolation stress for 2, 4, and 8 weeks are abnormal (Chen et al., 2016; Yu et al., 2018). The expression of ADAR1 in the frontal cortex and hippocampus of BALB/c mice exposed to chronic unpredictable stressors decreased significantly. After administration of ADAR1 inducer, the reduced expression of ADAR1 protein in the brain is recovered (Zhang et al., 2021a; Zhang et al., 2021b). In the mammalian genome, the ADAR family has three members: ADAR1, ADAR2, and ADAR3. Among them, ADAR1 and ADAR2 are active enzymes, while ADAR3 lacks enzyme activity (Song et al., 2016). ADAR1 has two splicing subtypes: ADAR1p110 and ADAR1p150. It catalyzes RNA editing by replacing adenosine with inosine (A-to-I editing) (Cho et al., 2017). ADAR1 plays an important role in maintaining the normal function of the nervous and immune systems (Veno et al., 2012; Nakahama and Kawahara, 2020). It has been reported that ADAR1 is involved in the pathogenesis of human diseases by editing and non-editing actions (Song et al., 2016; Gallo et al., 2017), including tumors (Nemlich Y et al., 2013; Han et al., 2015; Paz-Yaacov et al., 2015; Anadon et al., 2016; Chan et al., 2016), autoimmune diseases (Nakahama and Kawahara, 2020), cardiovascular diseases (Stellos et al., 2016), and mental diseases (Simmons et al., 2010; Kunii et al., 2011; Zhang et al., 2021a; Zhang et al., 2021b). Environmental stressors change the A-I editing mode catalyzed by ADAR1 (Simmons et al., 2010; Karanovic et al., 2015), thus affecting the expression of downstream target proteins. ADAR1 affects BDNF expression of the frontal cortex and hippocampus by acting on miR-432 and circ_0000418 in chronic unpredictable stress-treated mice (Zhang et al., 2021b). However, the mechanism of ADAR1 in chronic stress-induced cognitive dysfunction is still unclear.

In this study, we found that the administration of ADAR1 inducer (IFN-γ) alleviated the cognitive impairment of 3-week-old mice with chronic unpredictable stress exposure. These results suggest that ADAR1 is a key target molecule in the treatment of chronic stress-induced cognitive impairment. IFN-γ is closely linked to ADAR1. IFN-γ induces increased ADAR1 expression, which has been confirmed by several studies (Patterson and Samuel, 1995; Rabinovici et al., 2001; George et al., 2005; George et al., 2011; Zhang et al., 2020; Zhang et al., 2021a; Zhang et al., 2021b; Kim et al., 2021). The *in vitro* studies confirm that IFN-γ induces increased ADAR1 expression, including in human amnion U and SH-SY5Y cell lines (Patterson and Samuel, 1995), aluminum macrophages (MH-S cells) (Rabinovici et al., 2001), and mouse embryonic fibroblast (MEF) cells (George et al., 2005). In addition, the *in vivo* studies also confirm that IFN-γ reverses the decreased ADAR1 mRNA and protein expressions caused by chronic stress exposure (Zhang et al., 2020; Zhang et al., 2021a). The aforementioned studies are consistent with the results in this study, which show that as compared with the control group (C) PC12 cells, after IFN-γ treatment, ADAR1 inducer increased ADAR1 mRNA expression in PC12 cells of the IFN-γ treatment group (C + ADAR1 inducer) by 15%. *In vivo* study showed that as compared with that in the control group (C), after IFN-γ treatment, ADAR1 mRNA in the hippocampus and frontal cortex of mice in the ADAR1 inducer group (C + ADAR1 inducer) increased by 5% and 11%, respectively. As compared with that in the CUS model group (CUS), the expression of ADAR1 mRNA in the hippocampus of the IFN-γ treatment group (CUS + ADAR1 inducer) increased by 19%, the expression of ADAR1 protein in the hippocampus increased by 114%, the expression of ADAR1 mRNA in the frontal cortex increased by 22%, and the expression of ADAR1 protein in frontal cortex increased by 76%. The mechanism of IFN-γ inducing ADAR1 expression is as follows. It has been confirmed that ADAR1 transcription levels are increased by IFN-γ treatment measured by either northern hybridization or RT-PCR. The mouse Adar1 gene is composed of 15 exons, and its tissue structure is similar to that of the human ADAR1 gene (Liu et al., 1997; Hartner et al., 2004; Wang et al., 2004; George et al., 2005). Exon 1 of the ADAR1 gene appeared in three alternative forms: 1A, 1B, and 1C, respectively (George and Samuel, 1999a; Kawakubo and Samuel, 2000). The transcripts of exon 1A encode ADAR1p150 protein induced by IFN, and the transcripts of exon 1B or 1C (George and Samuel, 1999b) or exon 2 (Kawakubo and Samuel, 2000) encode constitutive ADAR1p110 protein (Kozak, 1989; Kim et al., 1994; Patterson and Samuel, 1995; Liu et al., 1997). It is known that under the intervention of IFN, the ADAR1 transcription level increases. ADAR1 transcription starts from multiple promoters, one of which can be induced by IFN (Patterson and Samuel, 1995; Liu et al., 1997; George and Samuel, 1999a). The IFN inducible promoter (PIA) has a 12 base pair (bp) IFN-stimulated response element (ISRE) with the characteristics of the type I IFN regulatory gene. PIA promoter induces kinase conserved sequence (KCS)-like elements in the upstream region of promoter ISRE (George and Samuel, 1999b; George et al., 2005). However, the relationship between ADAR1 and related downstream molecules of IFN-γ still need to be explored. IFN-γ is known to be involved in the pro-inflammatory response in microglia. IFN-γ impairs adult hippocampal neurogenesis and leads to depression-like behaviors and cognitive defects, which suggests that IFN-γ promotes neuron damage (Zhang et al., 2020). ADAR1 is supposed to be involved in neuron injury based on the fact that IFN-γ is ADAR1 inducer. In fact, ADAR1 has the dual effects of neuroprotection (Zhang et al., 2021a; Zhang et al., 2021b) and nerve injury (Zhang et al., 2020). It has been demonstrated that ADAR1 increases BDNF expression *via* miR-432. Maintaining ADAR1 homeostasis is the key mechanism to alleviate stress-induced dysfunction. Importantly, ADAR1 expression is impacted by genetic and environmental factors. It has been reported that ADAR1 expression in the brain of KM mice and BALB/c mice is increased and decreased after stress exposure, respectively (Chen et al., 2016; Yu et al., 2018). In addition, clinical studies have confirmed that IFN-α (it can induce increased ADAR1 expression) in the treatment of viral infections can induce some patients to have depression-like symptoms; however, some patients do not have depression-like symptoms (Pasquini M et al., 2008; Su KP et al., 2019). These results suggest that ADAR1 plays different roles in acute and chronic pathological states. Because of the dual role of ADAR1, our behavior results show that the mice in IFN-γ- or PEN-treated only groups show significant spatial and non-spatial cognitive dysfunctions as compared with that in the normal group due to breaking ADAR1 homeostasis. Meanwhile, ADAR1 expression decreases under stress exposure, which can be recovered by IFN-γ treatment.

In order to use ADAR1 as the target for reverse validation, PEN or EHNA was used as the inhibitor of ADAR1 in this study. PEN is a purine analogue isolated from *Streptomyces antibioticus* and is a potent inhibitor of adenosine deaminase (Sauter C et al., 2008). PEN works as an adenosine enzyme inhibitor, which may impact adenosine molecules catalyzed by the adenosine enzymes. Erythro-9-(2-hydroxy-3-nonyl)adenine (EHNA) was also used as an ADAR1 inhibitor (Cohen SS. 1985) in this study. It should be noted that EHNA is a powerful double inhibitor of adenosine deaminase and cyclic nucleotide phosphodiesterase 2 (PDE2). In the results shown in Figures 3D, G and Figure Fig4, EHNA was used as an ADAR1 inhibitor, so the effects on the inhibition of PDE2 also need to be considered. Being consistent with the published literature, the results of this study also confirmed that after the administration of EHNA, in PC12 cell lines, compared with that in control group C, the expression of ADAR1 mRNA decreased by 16%. In addition, *in vivo* study showed that compared with that in control group C, the expression of ADAR1 mRNA in the frontal cortex decreased by 12%, the expression of ADAR1 mRNA in the hippocampus decreased by 17%, and the expression of ADAR1 protein decreased by 23.3%. As compared with that in control group C, the expression of ADAR1 protein in the frontal cortex decreased by 25% after PEN administration. Compared with the model group (CUS), the expression of ADAR1 protein in the hippocampus of the EHNA intervention group (CUS + ADAR1 inhibitor) decreased by 7.6%, ADAR1 protein expression of the frontal cortex decreased by 7.9%, and ADAR1 protein expression of the frontal cortex decreased by 20.4% after PEN administration. However, it was unexpected that the expression of ADAR1 protein in the hippocampus increased by 86.7%, which may be related to the compensatory mechanism. At the same time, it seems to explain that in OLT results, compared with that in the CUS group, the discrimination index in the PEN treatment group (CUS + PEN) was significantly increased, and in ORT results, the discrimination index in PEN treatment group (CUS + PEN) showed increased tendency. It should be noted that ADAR1 knockout mice have a high mortality rate due to the important roles of ADAR1 (neurodevelopment and immunity function) in maintaining mice survival (Kim et al., 2021). So, ADAR1 knockout mice are unsuitable to endure stress exposure to be used in the study. Darcy et al. demonstrate that although IFN-γ knockout (KOS) mice show memory impairment in the basal state, these mice actually show better memory performance under chronic stress exposure (Litteljohn D et al., 2014). When the mice in basal state or chronic stress state were treated with ADAR1 inducer/inhibitor, the cerebral ADAR1 expression decreased or increased, which may have a dual effect on memory. In addition, we also agree with the view that IFN-γ is involved in the memory function changes caused by psychological stress with the related mechanism on brain monoamines activities (Litteljohn D et al., 2014).

**FIGURE 5:**
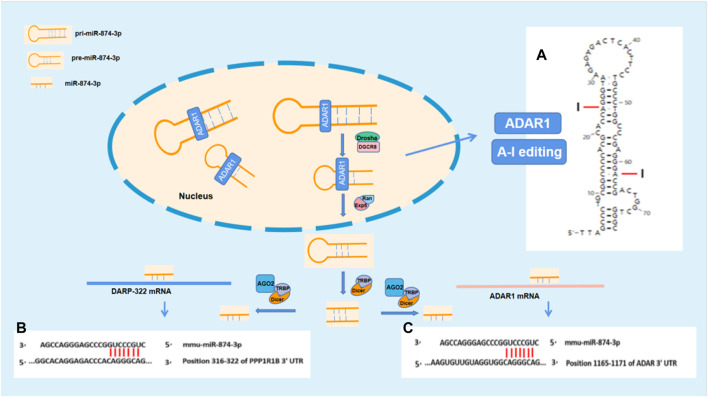
ADARl impacts DARPP-32 by acting on miR-874-3p. **(A)** The stem–loop structure of mmu-pre-miR-874 and the potential A-I editing sites; **(B)** and **(C)** show the potential binding sites for RNA interference between miR-874-3p and ADAR1 mRNA or DARP-32 mRNA, respectively. **(B)** Mmu-miR-874-3p contains seven sites binding to 3′UTR of ADAR mRNA; **(C)** mmu-miR-874-3p contains seven sites binding to 3′UTR of DARPP-32 mRNA.

In this study, we found that ADAR1 inducer alleviated the cognitive impairment and reversed the decreased DARPP-32 protein expression in the prefrontal cortex of chronic unpredictable stress-induced cognitive impairment mice. Further study showed that the expression of DARPP-32 mRNA and protein increased/decreased in PC12 cells with high/low expression of ADAR1. These findings suggest that ADAR1 is involved in the mechanism of stress-induced cognitive impairment *via* DARPP-32. DARPP-32, also known as PPP1R1B—phosphoprotein phosphatase 1 regulatory subunit 1B (Girault and Nairn, 2021) with an apparent relative molecular weight of 32,000, is a protein with thermal stability and acid stability composed of 200 amino acids. It is not only an inhibitor of phosphatase, such as protein phosphatase 1, but also an inhibitor of protein kinase, such as protein kinase A (Christensen and Nairn, 2021). DARPP-32 plays a two-way regulatory role in protein phosphorylation and dephosphorylation through its phosphorylation at different sites. DARPP-32 is distributed in neurons receiving dopaminergic projections in the brain (Walaas et al., 1983a). DARPP-32 is highly expressed in medium spinous neurons (MSN), independent of dopamine receptor subtypes (Walaas et al., 1983b; Beaulieu and Gainetdinov, 2011). It is also distributed in non-dopamine innervated cells (Aperia et al., 1990; Meister et al., 1991; Snyder et al., 1992). DARPP-32 phosphorylation is regulated by a variety of neurotransmitters and is at the center of the signal transduction pathway. It integrates a variety of intracellular signal transduction, regulates the electrical and chemical properties of neurons, regulates the physiological and behavioral responses of animals, and participates in a variety of physiological functions and pathological processes, including drug addiction, depression, and schizophrenia. DARPP-32 neuronal signals are known to be crucial for motivated behavior, learning, and memory (Walaas et al., 1983b). There are two main transcripts of DARPP-32: full-length (FL-DARPP-32) and truncated (t-DARPP-32). In autopsy samples, it was found that the expression of t-DARPP-32 increased in the prefrontal cortex of patients with schizophrenia and bipolar disorder, and the genotype with high expression of full-length DARPP-32 and low expression of truncated DARPP-32 has large volume of the prefrontal cortex. Larger volume of the prefrontal cortex is also associated with higher episodic memory performance, suggesting that differences in DNA sequence-related expression of DARPP-32 in frontal gray matter may lead to individual differences in episodic memory (Meister et al., 1991). These studies have confirmed that DARPP-32 plays an important role in cognitive function. However, it is not clear how ADAR1 participates in the pathogenesis of stress cognitive impairment by acting on DARPP-32. In order to explore the potential molecules involved in the impact of ADAR1 on DARPP-32, bioinformatics was performed with “TargetScanHuman.” The analysis results suggest that mmu-miR-874-3p are the potential molecules related to both ADAR1 and DARPP-32, which is confirmed by the results of this study, that is ADAR1 mRNA and DARPP-32 mRNA increased or decreased in PC12 cells with miR-874 high/low expression, respectively. It has been reported that ADAR1 inhibits the processing of mature miRNA by editing (A-to-I) the microRNA (miRNA) precursors (Nishikura et al., 2013, Nishikura, 2016). MiRNA synthesis was abnormal in ADAR1 knockdown HeLa cells (H. Ota et al., 2013). Compared with wild-type embryos, miRNA readings in ADAR1 −/− E11.5 embryos were significantly lower (H. Ota et al., 2013). A-I editing can affect the process of miRNA biogenesis, including Drosha cutting, Dicer cutting, RISC loading, and miRNA target selection (Nishikura et al., 2013, Nishikura, 2016). Further analysis also suggested that mmu-miR-874-3p have binding sites with ADAR1 mRNA and DARPP-32 mRNA. The stem–loop structures of mmu-pre-miR-874 were analyzed by RNAfold WebServer (http://rna.tbi.univie.ac.at//cgi-bin/RNAWebSuite/RNAfold.cgi). The pre-mRNAs of mmu-pre-miR-874 have potential sites edited by A-to-I. Our results suggest that ADAR1 is supposed to act on pri-mRNA or pre-mRNA of miR-874-3p *via* A-to-I editing, affect the bio-production of mature miR-874-3p, and then impact DARPP-32 expression *via* RNA interference, which is involved in the mechanism of stress-induced cognitive impairment (Figure 5).

In summary, the results of this study demonstrate that ADAR1 affects the expression of DARPP-32 *via* miR-874-3p, which is involved in the molecular mechanism of pathogenesis in chronic unpredictable stress-induced cognitive impairment. The new findings of this study provide a new therapeutic strategy for the prevention and treatment of stress cognitive impairment from epigenetics. The limitation of this study is that the animal model of ADAR1 over-expression in specific brain areas, such as the hippocampus or frontal cortex, will be helpful to reconfirm the role of ADAR1 in cognition-related brain areas. The RNA editing mechanism of ADAR1 acting on DARPP-32 mRNA precursor also need to be further explored in the future.

## Data Availability

The original contributions presented in the study are included in the article/Supplementary Material; further inquiries can be directed to the corresponding author.

## Glossary

ADAR: adenosine deaminase acting on RNA
A-to-I RNA editing: adenosine to inosine RNA editing
BDNF: brain-derived neurotrophic factor
CUS: chronic unpredictable stress
CG: normal control group
CG + IFN-γ: ADAR1 inducer intervention control group
CG + PEN: ADAR1 inhibitor intervention control group
CUS + IFN-γ: ADAR1 inducer intervention model group
CUS + PEN: ADAR1 inhibitor intervened in model group
CDK5: cyclin-dependent-like kinase 5
CSNK2B: casein kinase II subunit beta
DARPP-32: protein phosphatase 1 regulatory subunit 1B
EP: Eppendorf
Hs: *Homo sapiens*
IFN-γ: interferon-gamma
mPFC: medial prefrontal cortex
Mmu: *Mus musculus*
miR: microRNA
ORT: new object recognition test
OLT: new object localization test
PBS: phosphate buffer saline
PVDF: polyvinylidene fluoride
PMSF: phenylmethanesulfonyl fluoride
PPP2R1A: serine/threonine-protein phosphatase 2A 65 kDa regulatory subunit A alpha isoform
PPP2R5D: serine/threonine-protein phosphatase 2A 56 kDa regulatory subunit delta isoform
PPP3CA: serine/threonine-protein phosphatase 2B catalytic subunit alpha isoform
PPP3R1: calcineurin subunit B type 1
PPP1CC: serine/threonine-protein phosphatase PP1-gamma catalytic subunit
PRKAR2B: cAMP-dependent protein kinase type II-beta regulatory subunit
PRKACA: cAMP-dependent protein kinase catalytic subunit alpha
PRKACB: cAMP-dependent protein kinase catalytic subunit beta
PPP2CB: serine/threonine-protein phosphatase 2A catalytic subunit beta isoform
PPP2CA: serine/threonine-protein phosphatase 2A catalytic subunit alpha isoform
PPP1CA: serine/threonine-protein phosphatase PP1-alpha catalytic subunit
